# Human activity accelerating the rapid desertification of the Mu Us Sandy Lands, North China

**DOI:** 10.1038/srep23003

**Published:** 2016-03-10

**Authors:** Yunfa Miao, Heling Jin, Jianxin Cui

**Affiliations:** 1 Key Laboratory of Desert and Desertification, Cold and Arid Regions Environmental and Engineering Institute, Chinese Academy of Sciences, Lanzhou 730000, China; 2 Northwest Institute of Historical Environment and Socio-Economic Development, Sha’anxi Normal University, Xi’an 710062, China

## Abstract

Over the past several thousand years, arid and semiarid China has experienced a series of asynchronous desertification events in its semiarid sandy and desert regions, but the precise identification of the driving forces of such events has remained elusive. In this paper we identify two rapid desertification events (RDEs) at ~4.6 ± 0.2 ka BP and ~3.3 ± 0.2 ka BP from the JJ Profile, located in the eastern Mu Us Sandy Lands. These RDEs appear to have occurred immediately following periods marked by persistently frequent and intense fires. We argue that such fire patterns, directly linked to an uncontrolled human use of vegetation as fuel, played a key role in accelerating RDEs by ensuring that the land surface was degraded beyond the threshold required for rapid desertification. This would suggest that the future use of a massive and sustained ecological program of vegetation rehabilitation should reduce the risk of destructive fire.

The arid and semiarid region of North China consists principally of sandy lands, deserts and loess deposits, and lies at the junction of four principal atmospheric circulatory systems (Fig. 1A). Under global warming the risks of desertification and humidification in the sandy areas are carefully evaluated based on the parameters obtained from written records and observational data^1^,^2^ as well as longer time-series proxies of sedimentary materials (*e.g*., grain-size^3^,^4^,^5^, pollen^6^,^7^,^8^ and black carbon^9^ *etc*.), because all of these sources can offer opportunities for understanding the historic relations among desertification, climate and human activities, and thus serve as references for major ecological rehabilitation. More reliable modern research shows that RDEs during the past 10 ka occurred asynchronously in different sandy lands, or even different parts of the same sandy lands^10^,^11^; although the driving forces behind these events remain elusive, they most probably include climatic^10^,^12^,^13^ and human^14^ factors, or a combination of both^1^,^11^,^15^. However, the reduced precipitation resulting from a weakening Asian monsoon was thought to be the principal factor controlling green/sandy shifts in these areas^6^,^7^,^8^,^9^.

Here we report on a Mid to Late Holocene microcharcoal record used as a proxy for fire characteristics in the Mu Us Sandy Lands (Fig. 1B) in an attempt to explore the relation between fire and RDEs. This allows us to offer some suggestions regarding the future ecological management of sandy areas in the Mu Us Sandy Lands.

## Materials

**Figure 1:**
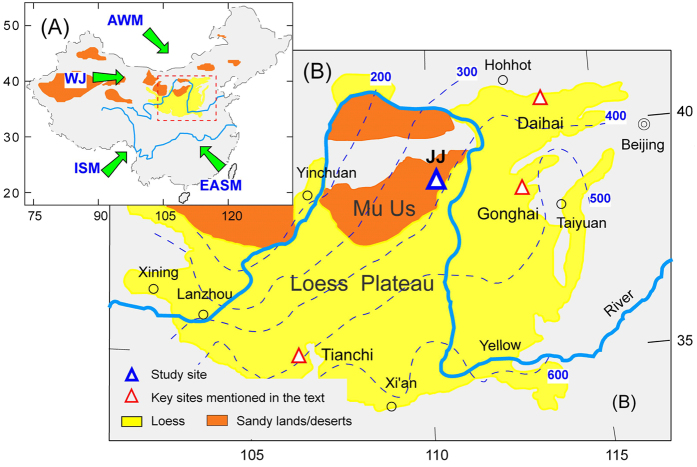
(**A**) Major atmospheric circulation regimes in East Asia and (**B**) the geographical location of the Mu Us Sandy Lands, including the location of the JJ Profile. Dashed lines denote mean annual precipitation isohyets. ISM: Indian Summer Monsoon; EASM: East Asian Summer Monsoon; EAWM: East Asian Winter Monsoon, WJ: Westerly jetstream. Hollow red triangles show the locations of Daihai Lake^3^,^6^,^9^, Gonghai Lake^7^ and Tianchi Lake^8^. The map was generated by the software Adobe^®^ FreeHand MX.

The Mu Us Sandy Lands are surrounded by the Yellow River to the west, north and east, and are adjacent to the neighboring Chinese Loess Plateau (CLP) to the southeast (Fig. 1A). The region is dominated by a typical arid/semiarid continental monsoonal climate, with a mean annual temperature of 6~9 °C and mean annual precipitation of 200~400 mm. Mean annual evaporation is 1800~2500 mm. In winter, the prevailing northwesterly winds near the surface play an important role in defining the most powerful sandstorm pathways (Fig. 1B). Temperate desert steppe and steppe dominate this area, dominated by *Artemisia*, *Caragana*, *Salix*and *Hippophae*.

**Figure 2:**
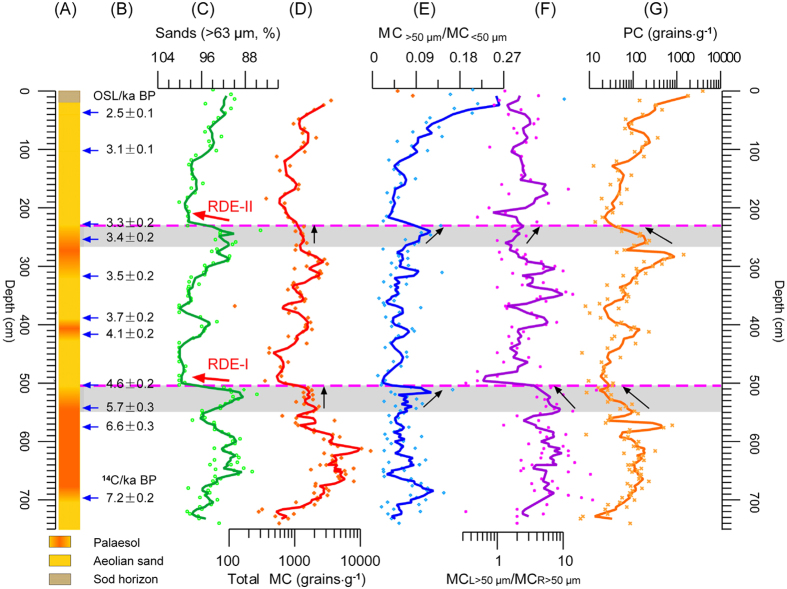
Comprehensive correlations in (**A**) lithology, (**B**) dating, (**C**) sand (>63 μm) content, (**D**) total MC, (**E**) MC_>50 μm_/MC_<50 μm_, (**F**) CC_L>50 μm_/CC_<50 μm_ and (**G**) total PC in the Mu Us JJ Profile. All curves are smoothed using a 3-point running average. Gray rectangles show the two periods of abnormal fire patterns prior to the RDEs. Horizontal dotted lines show the boundaries of the RDEs. Black arrows show direction of trends. RDEs: rapid desertification events. MC: microcharcoal concentration. PC: pollen concentration.

The JJ Profile (38^o^44.594′N, 110^o^10.044′E; 1159 m above sea level) is located in the second terrace of the Tuwei River, a branch of the Yellow River, on the eastern edge of the Mu Us Sandy Lands. The profile is 740 cm deep; from the base upwards it is stratigraphically divided into paleosol, eolian sand, weakly-developed paleosol and gramineous layers (Fig. 2A). Strata at ~510 cm and ~230 cm are correlated with two RDEs (RDE-I, ~4.6 ± 0.2 ka BP; RDE-II, ~3.3 ± 0.2 ka BP (Fig. 2B), respectively) characterized by ~100% coarse sand quartz (>63 μm) (Fig. 2C).

Microcharcoals were extracted using a standard pollen methodology (SI text and Fig. S1). A known number of *Lycopodium clavatum* spores were initially added to each sample for calculating the microcharcoal concentration (MC). Additionally, four grain-size groups (<30 μm, 30–50 μm, 50 μm and >100 μm), and two shape types (sub-round (R) and sub-long (L)), were identified. Here, total MC (Fig. 2D), MC_>50 μm_/MC_<50 μm_ (Fig. 2E) and MC_L>50 μm_/MC_R>50 μm_ (Fig. 2F) ratios were obtained to infer fire strength (frequency and/or intensity), fire travel distance (open and/or local) and fire material types (herbaceous and/or woody), respectively; additionally, the total pollen concentration (PC) was employed to assess vegetation coverage (Fig. 2G).

## Results and Discussion

Total MC varies between ~30 and ~3200 grains·g^−1^, with an average of ~940 grains·g^−1^ (Fig. 2D), and maintains a roughly opposing pattern to that of the sand content (grain-size of >63 μm). At the same time, the MC_>50 μm_/MC_<50 μm_ ratio remains at a stable value of ~0.07 except for in the uppermost part of the Profile (where it reaches ~0.27) (Fig. 2E). The MC_L>50 μm_/MC_R>50 μm_ ratio varies between 32.0 and 0.3, with a mean of 4.8 (Fig. 2F). The PC ranges roughly between 3912 and 6 grains·g^−1^, with a mean of ~154 grains·g^−1^; it follows a pattern that is roughly similar to that of total MC (Fig. 2G).

According to the assemblages, three distinct periods of fire strength versus vegetation cover are identified. Before 4.6 ± 0.2 ka BP (Stage I), the relation between fire and vegetation evinces a ‘bow’ shape pattern. Between 4.6 ± 0.2 ka BP and 3.3 ± 0.2 ka BP (Stage II), this relation exhibits a gentle, similarly increasing trend. After 3.3 ± 0.2 ka BP (Stage III), the relation between fire and vegetation follows a similar pattern to Stage II, though with a sharp increase in distances traveled by fires toward the end of the period (Fig. 2E).

### Fire trends and climate change

As the residual matter from the incomplete burning of vegetation, microcharcoal is usually considered as the most reliable natural proxy for assessing the intensity and strength of fires^16^,^17^,^18^. The singular wind-blown nature of the JJ Profile indicates that, here at least, total MC is indeed likely to be a good proxy for the intensity and frequency of any fires^16^,^17^,^19^,^20^,^21^; the relatively larger grains tend to travel a shorter distance and reflect local fire characteristics^21^, and are associated with a high MC_>50 μm_/MC_<50 μm_ ratio; the sub-long and sub-round shapes of grains are linked to vegetation types, and in particular grasses and woody plants^22^, with a greater MC_L>50 μm_/MC_R>50 μm_ ratio indicating a relatively greater proportion of grasses. At the same time, a higher PC can indicate dense vegetation cover (or vegetation mass) under a moister climate, especially when located under a single wind-blown sedimentary layer^23^,^24^.

During Stage I, the ‘bow’ shape of the vegetation cover versus fire strength time series correlated well with the reconstructed precipitation record based on pollen data from lakes Tianchi and Gonghai^7^, indicating a correspondingly wetter climate during the Mid Holocene. The chemical proxies (Ru/Sr, CIA *etc*.) in the JJ Profile further supported such a climatic pattern^5^. The black carbon content from Daihai Lake also experienced a ‘bow’-shaped trend, with strong fluctuations^9^, attributed to the result of the increasing vegetation cover under an improving climate, leading to more vegetation being available for combustion^9^,^25^.

### Fire aberrances before RDEs

**Figure 3:**
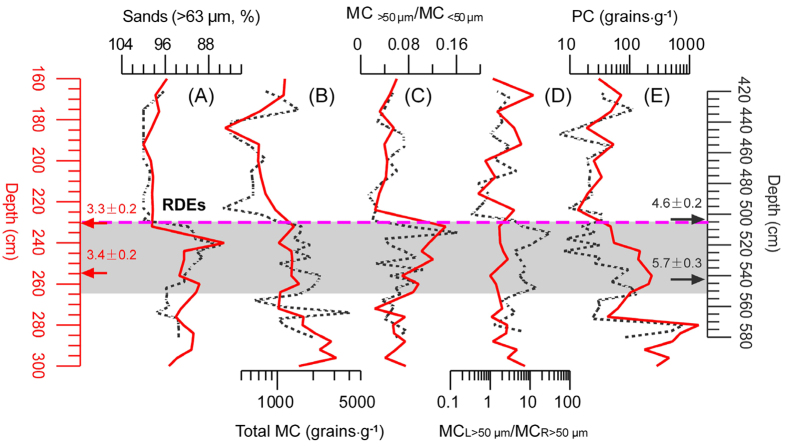
Comparisons between (**A**) sand content, (**B**) total MC, (**C**) the MC_>50 μm_/MC_<50 μm_ ratio, (**D**) the MC_L>50 μm_/MC_R>50 μm_ ratio and (**E**) PC across RDE-I (continuous red line) and RDE-II (dashed black line) in the JJ Profile, Mu Us Sandy Lands.

When looking at the records in more detail, total MC, MC_>50 μm_/MC_<50 μm_, MC_L>50 μm_/MC_R>50 μm_ and PC before the two RDEs exhibit particular characteristics (Fig. 3): both total MC records maintain stable values between ~1000–1100 grains·g^−1^; the MC_>50 μm_/MC_<50 μm_ ratios increase obviously from ~0.08 to ~0.16; the MC_L>50 μm_/MC_R>50 μm_ ratios evince clear gaps (~2 in RDE-I and ~10 in RDE-II); and total PC decreases gradually from ~200 to ~20 grains·g^−1^.

A dry climate and frequent strong winds promote desertification. One classic explanation of the above results is the influence of nonlinear relations combined with feedback between desertification and the atmosphere^12^,^21^,^26^,^27^. However, the most recent results support the view that atmospheric change seem to occur contemporaneously and widely, meaning that any sediments extant under such an atmosphere can exhibit similar responses. For example, millennial-scale Younger Dryas and Heinrich events are well-recorded by coarser grain-sizes on the CLP, where such sediments were transported further, and deposited by, the stronger winds resulting from a cooler drier climate^4^. This suggests that any Holocene dry events should also be contemporaneous with changes in sedimentary characteristics under the similar atmospheric dynamics^28^. Regardless of any nonlinear^12^,^26^,^27^ or linear relations^6^,^7^, desertification in North China has tended to occur under a cool/dry climate^1^,^4^,^10^,^11^.

However, through careful comparison, these two RDEs at 4.6 ± 0.2 ka BP and 3.3 ± 0.2 ka BP cannot be directly attributed to climate change, owing to the following four key contradictions. (i) These RDEs occur synchronously in the JJ Profile and at Daihai Lake^3^, which is difficult to dismiss as merely a coincidence. Because at Daihai Lake, RDE-II occurs during a period which is characterized by a rapid decrease in tree pollen, whereas RDE-I occurs when tree pollen percentages are high. These two RDEs cannot both therefore be explained as being the result of a climatic deterioration (cooling and drying) in the climate^6^. Additionally, at Lakes Gonghai^7^ and Tianchi^8^, the climate begins to deteriorate at 5.3 ka BP, but no obvious rapid climate change events occur during that time. (ii) Total MC and PC trends follow different directions, whereas according to the precipitation data from Stage I, they should have declined similarly along with the deterioration in the climate. (iii) The relatively local fires become consistently stronger, meaning that the area proximate to the JJ Profile experienced the burning of greater quantities of vegetation, rather than the lesser quantities of vegetation that would be expected were the climatic deteriorating. (iv) There exist sharply different fire abnormality timespans prior to these two RDEs: over 1000 years versus 100 years, and under relatively ‘better’ and ‘worse’ vegetation/climate conditions, respectively. If these RDEs were merely controlled by climate abnormity, they might exhibit similar timespans *vis-à-vis* millennial-scale oscillations^28^,^29^,^30^,^31^.

### Human activities driving RDEs

We therefore propose that the aberrant patterns of fire intensity/strength exhibited prior to both RDEs are the direct result of human activity, *i.e.* the excessive burning of vegetation for fuel, and an accelerating land surface degradation beyond the RDE threshold. Humans exert a definitive impact upon the environment, as when they alter terrestrial vegetation patterns during the rapid development of agriculture, and when a sharp increase in the population necessitates significant changes in land use^32^. This is especially marked when considerable quantities of vegetation are burnt for fuel^33^,^34^. Under such a scenario, the vegetation coverage and local environment may be significantly altered by “slash and burn” agricultural activity, rural construction, and the use of fire for producing pottery, cooking and providing warmth^34^.

The Mu Us Sandy Lands and their neighboring regions have been intensively inhabited since at least the Last Glacial Maximum^35^. Human agriculture flourished during the Holocene, especiallyin cultural periods such as the Yangshao, Longshan, Zhukaigou and Maoqinggou (~9.0–2.0 ka BP)^36^,^37^,^38^,^39^. Over 400 ancient cultural sites dating to before 4.0 ka BP have been identified in the Mu Us Sandy Lands^40^. Additionally, several fire events appear to have occurred in the regions surrounding the Mu Us Sandy Lands, based on charcoal assemblages^34^ and total MC^8^,^21^ and black carbon content^9^ values. These show multiple periods of human activity at different sites during the Mid to Late Holocene. For example, the Shimou archeological site in the Mu Us Sandy Lands, ~20 km to the south of the JJ Profile, has been listed in the world’s top ten ‘Ten Amazing Cities from the Ancient World’ (http://www.ancient-origins.net). Covering over four square kilometers, this stone city, with numerous carved jade and mural fragments, indicates the existence of a very prosperous urbanized society at ~4 ka BP.

## Discussion and Conclusion

**Figure 4:**
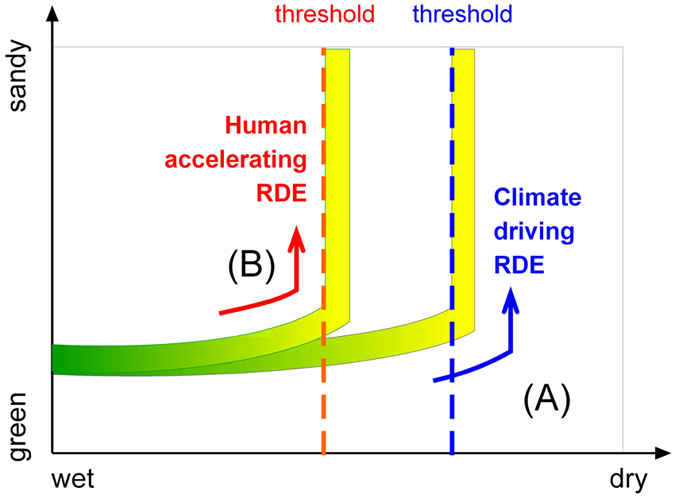
RDE threshold model for the Mu Us Sandy Lands based on responses to climate change and human activity indicated by fire records. (**A**) As precipitation decreases, the system moves towards a drier state, resulting in a crossing of the RDE threshold^12^. (**B**) After superimposing human activity, under a background of decreasing precipitation, we encounter the irreversible destruction of vegetation beyond the RDE threshold, when vegetation is used for fuel.

After considering the relations between RDEs, climate change and human activity in the Mu Us Sandy Lands, we outlined our RDE trigger models. Based simply upon the deterioration in the climate, the lands system moves towards a drier state until it reaches the RDE threshold (Fig. 4A); this can reasonably be applied to an area such as the Hunshandake Sandy Lands before groundwater extraction is taken into account^12^. After superimposing human activity, under the same climatic process, the barren land surface caused by human activity can accelerate any risk that the RDE threshold is crossed (Fig. 4B). In the historic Mu Us Sandy Lands this finding is coincident with the data from many Neolithic and Bronze Age archeological sites in this region^36^,^38^,^40^ (Fig. S7).

Today, the Mu Us Sandy Lands are an important agro-pastoral transition zone and a powerful ecologically-protective screen preventing the incursion of deserts from the south and east. According to this analysis, future major ecological rehabilitation should focus on improving vegetation coverage and avoiding fire hazards to reduce the risk of desertification. Once the vegetation has been seriously damaged, land degeneration will greatly increase the risk that the RDE threshold is crossed.

## Additional Information

**How to cite this article**: Miao, Y. *et al.* Human activity accelerating the rapid desertification of the Mu Us Sandy Lands, North China. *Sci. Rep.* **6**, 23003; doi: 10.1038/srep23003 (2016).

## Supplementary Material

Supplementary Information
