# Supplementary material for: Human activity accelerating the rapid desertification of the Mu Us Sandy Lands, North China
Source: Sci Rep. 2016 Mar 10;6:23003. doi: 10.1038/srep23003 (PMC4785332; doi:10.1038/srep23003)
Supplement: Supplementary Information [file srep23003-s1.doc]

**Supplementary Information**

**Human activity accelerating the rapid desertification of the Mu Us Sandy Lands, North China**

Yunfa Miao ^1^*, Heling Jin ^1^, Jianxin Cui ^2^

**Supplementary Text**

**Materials and Methods**

*Microcharcoal and pollen collection*

For microcharcoal and pollen extraction, 103 samples of 25-30g mass were taken at 2-6cm intervals and extracted using a standard pollen methodology, *i.e.* acid digestion (treatment with 10% HCl and 40% HF acid to remove carbonates and silicates, respectively), followed by fine sieving, to enrich the microcharcoals and pollen grains. The prepared specimens were mounted in glycerol for identification. Each sample was counted under a light microscope at 400× magnification at regularly-spaced traverses. Microcharcoal and pollen grains were counted and photographed using a Leica DM4000 B microscope; some typical samples were examined under a MLA 650 scanning electron microscope (SEM) (Fig. S1). All samples were studied at the Cold and Arid Regions Environmental and Engineering Research Institute, Chinese Academy of Sciences (CAS). A known number of *Lycopodium clavatum* spores (batch #27600) were initially added to each sample for calculating the MC and PC^S1^. Total MC was calculated using the following formula:

MC=*N_n_*/*L_n_*×27600*/W_n_*

where *N* is the identified number of microcharcoals; *L* is the number of *Lycopodium clavatum*; *W* is the dried weight of the sample; and *n* is the sample number.

The microcharcoals were divided into four grain-size groups: <30 μm, 30-50 μm, 50 μm and >100 μm. These were defined using the long axes of each individual grain. At the same time, two shape types, sub-round (R) and sub-long (L) were identified being calculated using the long axis to short axis ratio, <2.5 versus >2.5, respectively. MC_R<30 μm_, MC _R30-50 μm_, MC _R50-100 μm_, MC_R >100 μm_, MC_L<30 μm_, MC _L30-50 μm_, MC _L50-100 μm_, MC_L >100 μm_ refer to the respective MC with related shapes and sizes (Fig. S2, Table S3).

**Supplementary Figures**

**
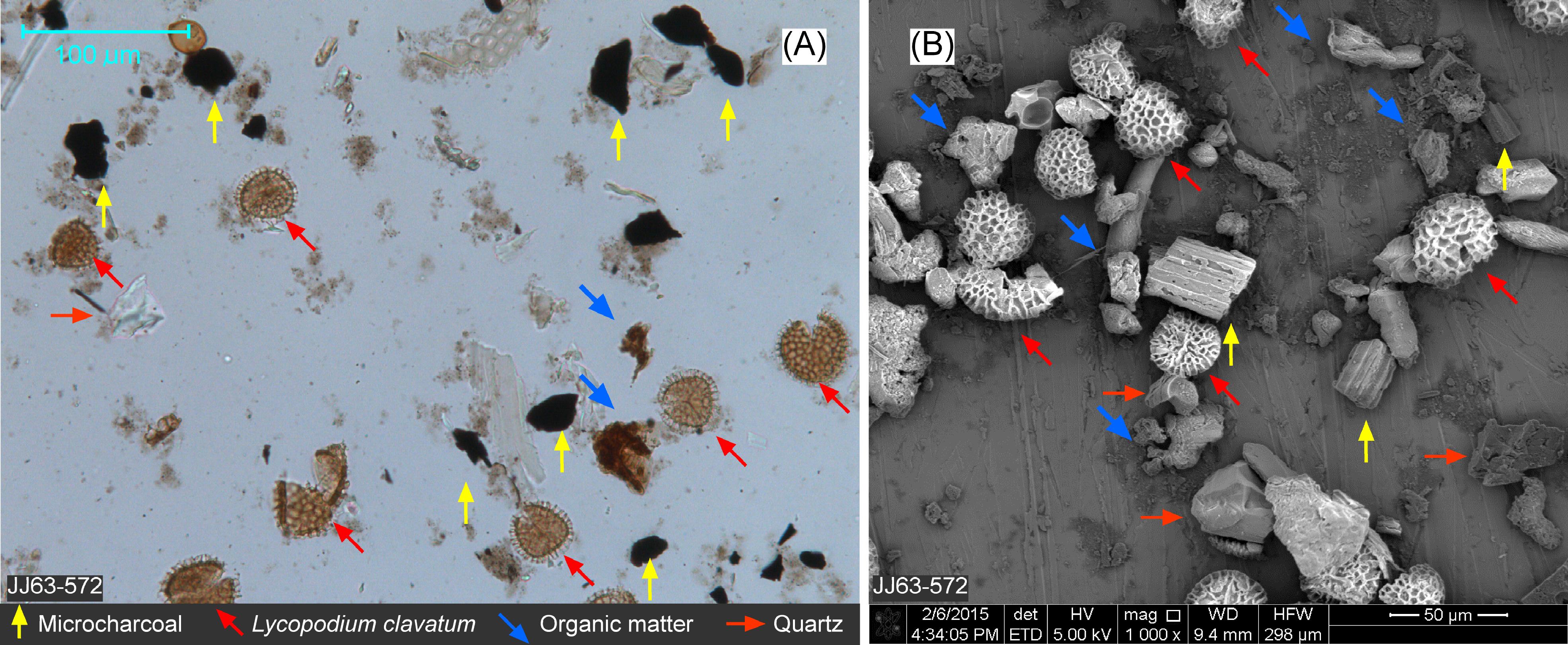
**

**Fig. S1.** View of the palynofacies of the JJ Profile under (A) the microscope, and (B) the Electronic Scanning Microscopic (ESM). letters plus number in the bottom left represent the sample number.

**
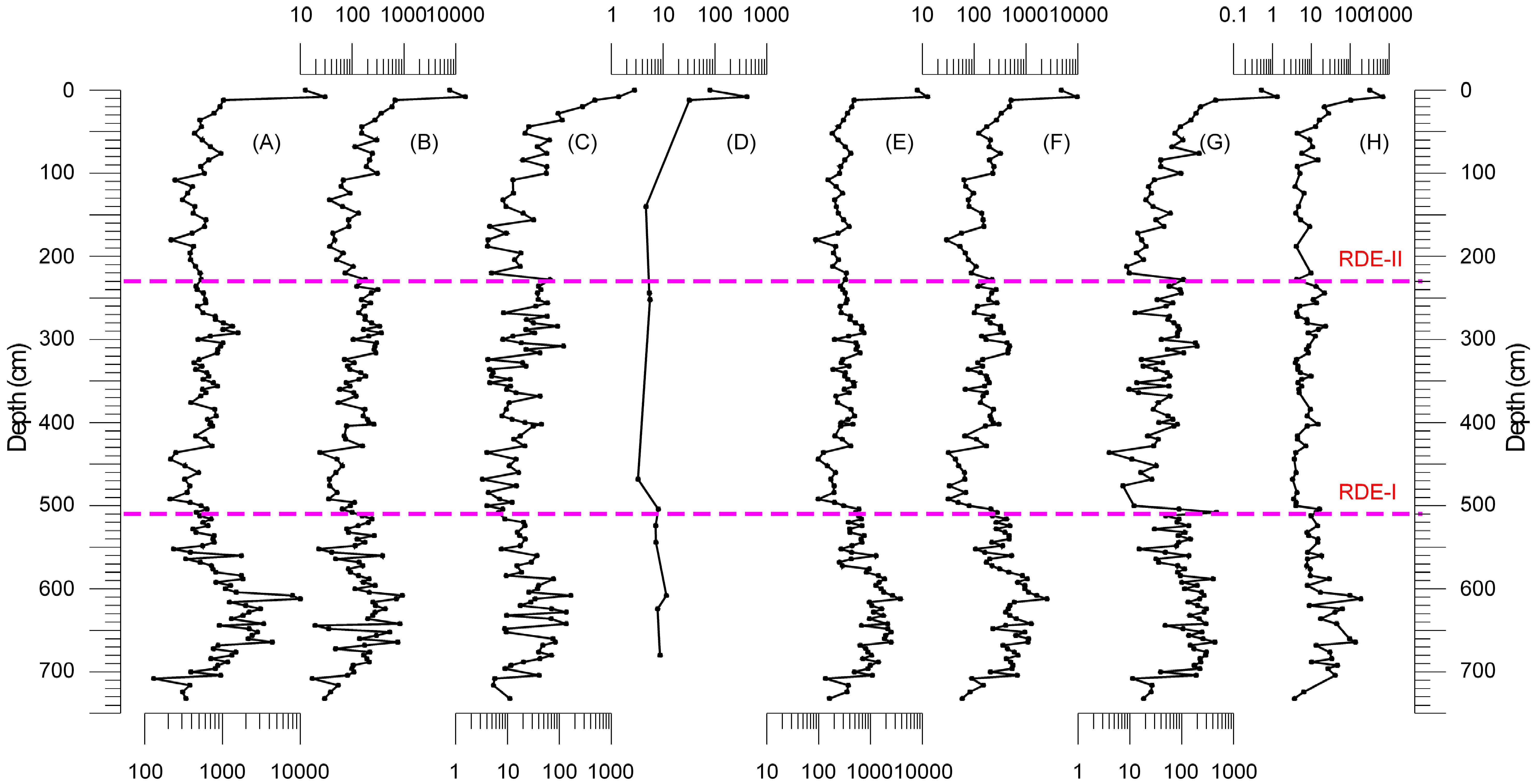
**

**Fig. S2.** The sub-round MC records for (A) <30 μm, (B) 30-50 μm, (C) 50-100 μm, (D) >100 μm and sub-long MC records for (E) <30 μm, (F) 30-50 μm, (G) 50-100 μm and (H) >100 μm values in the JJ Profile, Mu Us Sandy Lands (unit: grains·g^-1^).

**
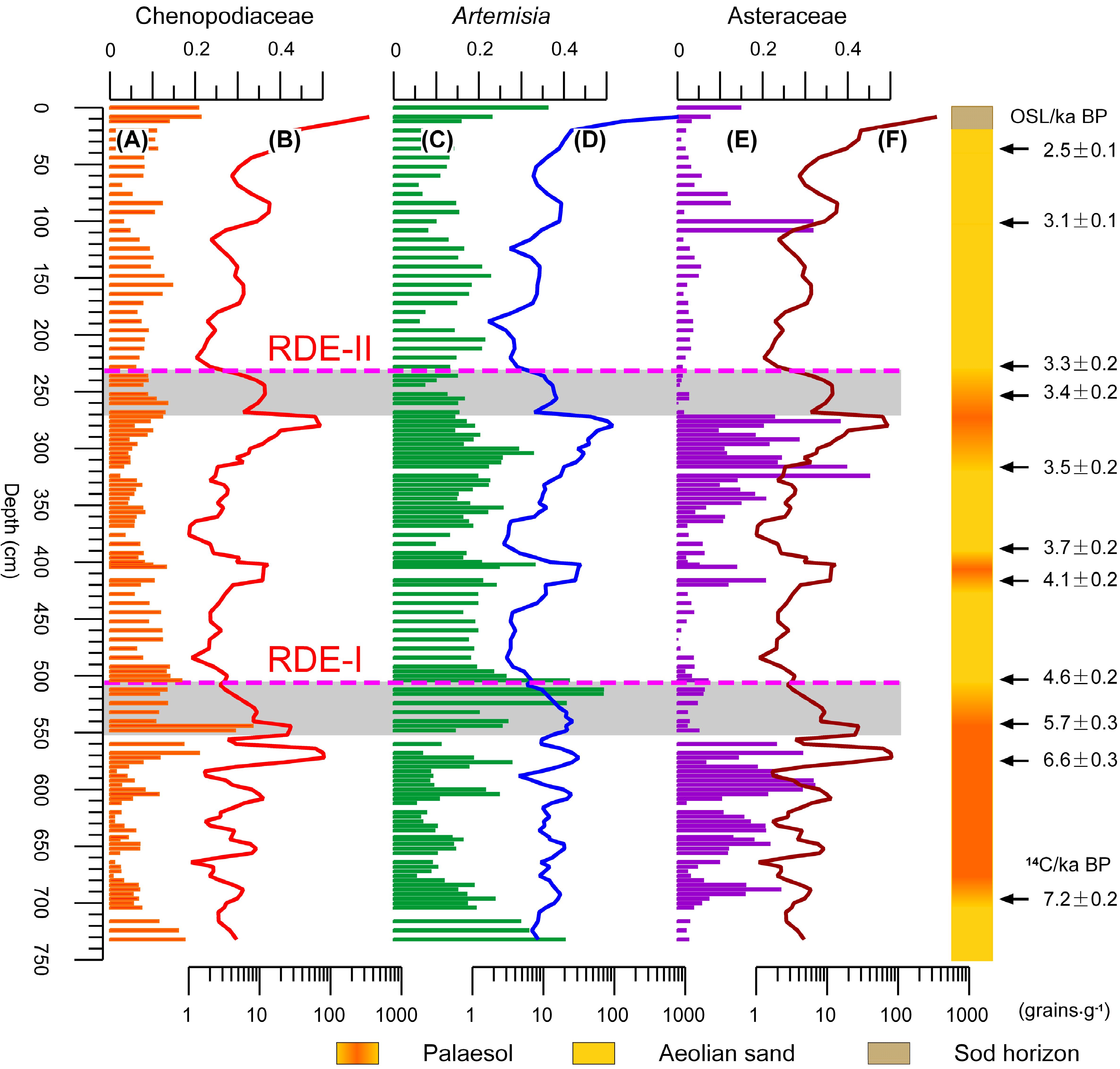
**

**Fig. S3.** Diagram of (A, C, E) selected pollen relative contents compared to (B, D, F) related pollen concentrations (units: grains·g^-1^) for the JJ Profile, Mu Us Sandy Lands. Asteraceae content decreases sharply before both RDEs.

**
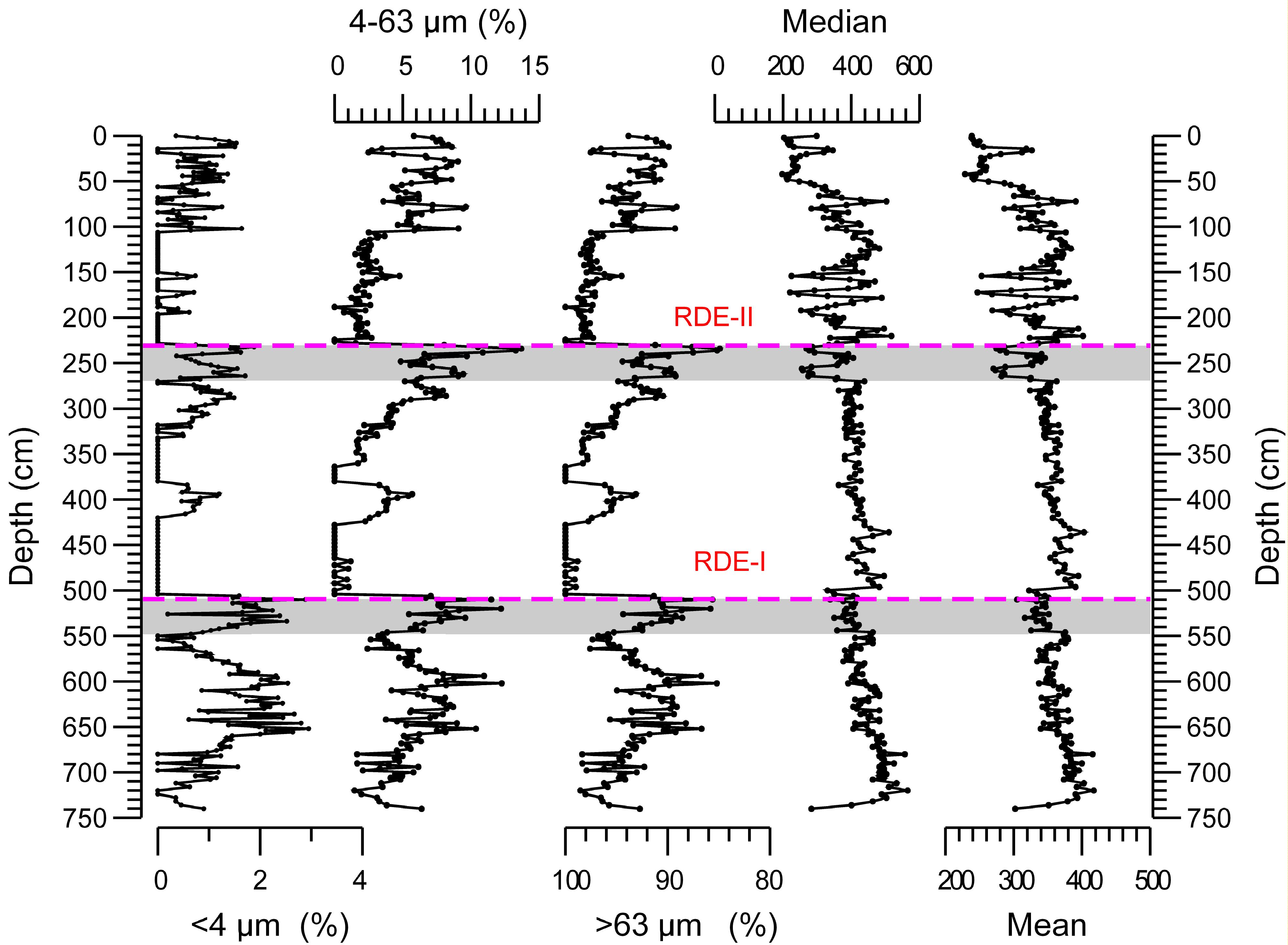
**

**Fig. S4.** The grain size of [quartz](javascript:void(0);) [sand](javascript:void(0);) distribution record for the JJ Profile, Mu Us Sandy Lands.

**
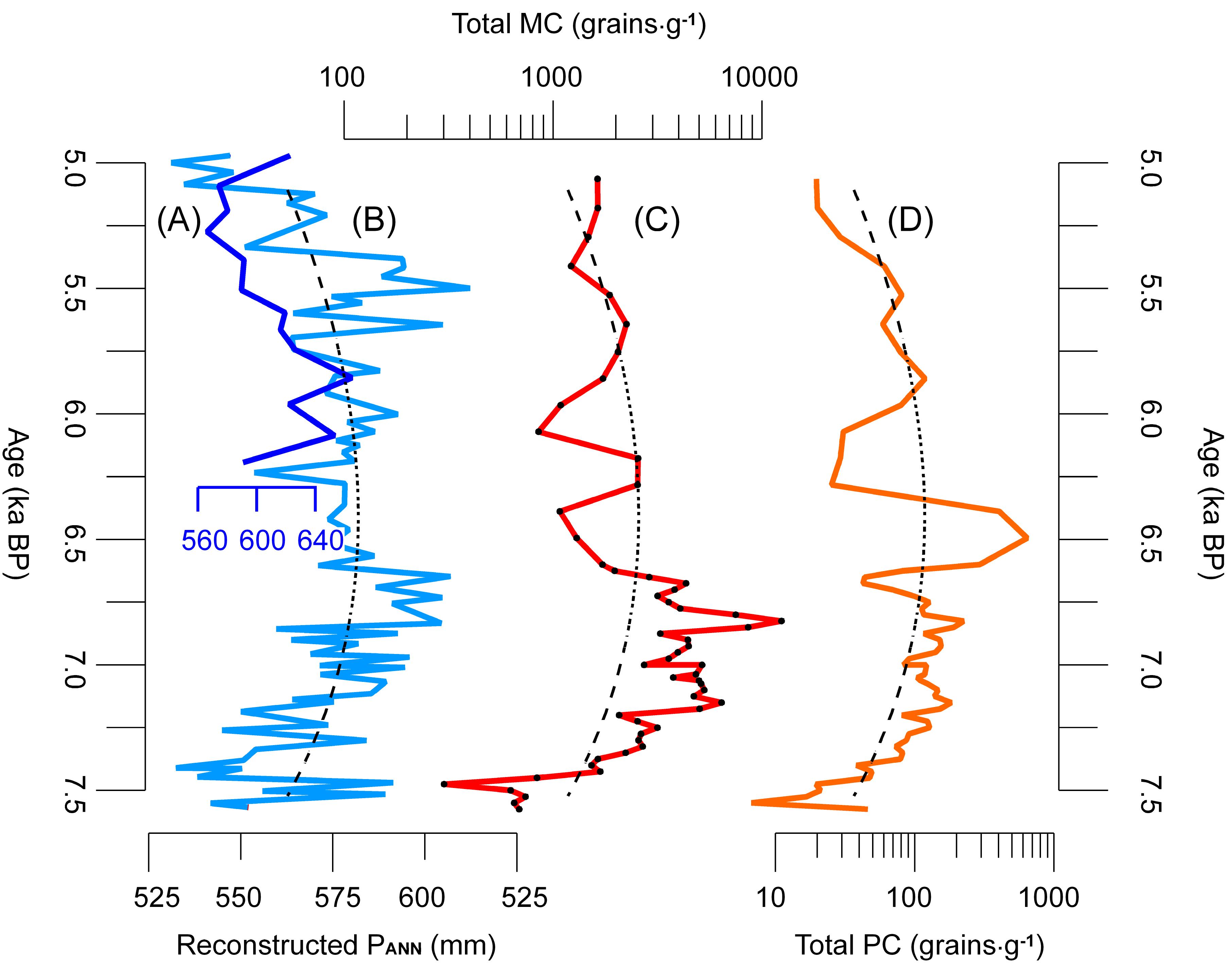
**

**Fig. S5.** Correlations between (A) pollen-based annual precipitation (P_ANN_) at Tianchi Lake (*8*) and (B) P_ANN_ at Gonghai Lake (*7*) and (c) MC and (D) PC values from the JJ Profile (this study). The JJ Profile was dated using linear interpolation.

**
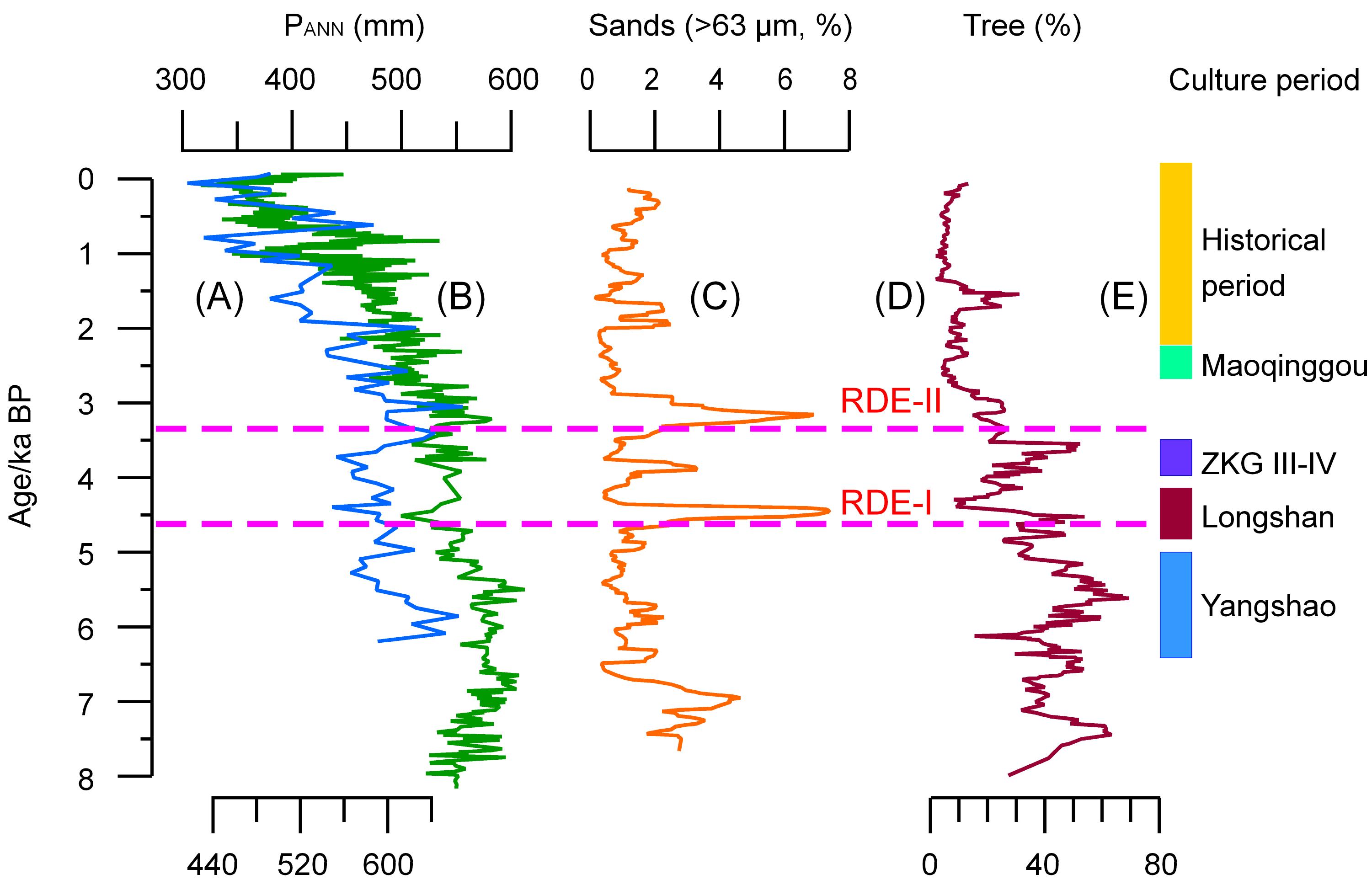
**

**Fig. S6.** Correlations between precipitation states, RDEs and cultural periods. (A) Pollen-based annual precipitation (P_ANN_) from Gonghai Lake (*7*) and (B) P_ANN_ from Tianchi Lake (*8*); (C) sand (>63 μm) content (using a 7-point running mean) (*3*), (D) tree pollen percentages from Daihai Lake (*6*), and (E)the archeological records of the cultural periods of the Daihai Lake region (*9*), including the Yangshao culture (6.5-5.0 ka BP), Longshan culture (4.6-4.0 ka BP), Zhukaigou III-IV cultural periods (~4.0-3.6 ka BP), and the Maoqinggou culture extant during the Ordos Bronze Age period (~2.6-2.3 ka BP).

**
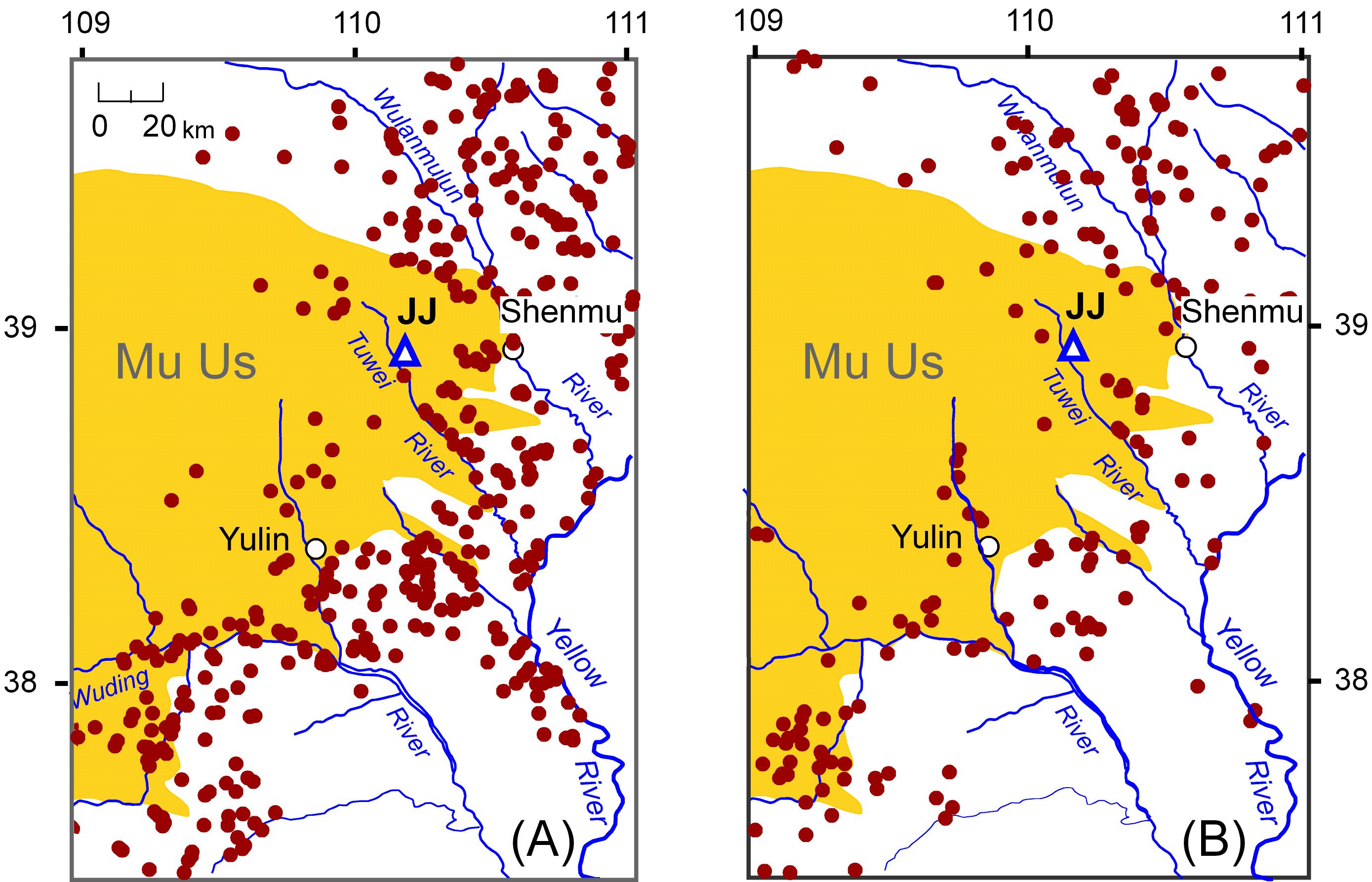
**

**Fig. S7.** Ancient cultural sites in the eastern Mu Us Sandy Lands (A) 9-4 ka BP and (B) 4-2 ka BP (after ref. *40*). Massive human activity has occurred in the Mu Us Sandy Lands since the Early to Mid Holocene. The map was generated by the software Adobe® FreeHand MX.

**Supplementary Tables**

**Table S1.** OSL dating results and related parameters from the JJ Profile, Mu Us Sandy Lands (*5*).

| Lab  No. | Sample No. | Depth  (cm) | Grain size  (um) | U (ppm) | Th (ppm) | K (%) | D*e*  (Gy) | Dose rate  (Gy/ka) | Water content  (%) | OSL/ka |
| --- | --- | --- | --- | --- | --- | --- | --- | --- | --- | --- |
| 13G-90 | JJ-19 | 38 | 4~11 | 0.64 | 2.32 | 2.33 | 7.30±0.25 | 2.96±0.12 | 5±1 | 2.5±0.1 |
| 13G-91 | JJ-51 | 102 | 4~11 | 0.81 | 3.59 | 1.93 | 8.42±0.18 | 2.73±0.11 | 5.5 | 3.1±0.1 |
| 13G-92 | JJ-114 | 228 | 4~11 | 0.66 | 3.39 | 2.26 | 9.71±0.50 | 2.97±0.12 | 5±1 | 3.3±0.2 |
| 13G-93 | JJ-126 | 252 | 4~11 | 0.70 | 3.44 | 2.44 | 10.80±0.26 | 3.16±0.13 | 5.7 | 3.4±0.2 |
| 13G-94 | JJ-159 | 318 | 4~11 | 0.53 | 1.75 | 2.40 | 10.02±0.31 | 2.89±0.12 | 3.4 | 3.5±0.2 |
| 13G-95 | JJ-181 | 388 | 4~11 | 0.58 | 2.38 | 2.34 | 10.75±0.40 | 2.89±0.12 | 4.2 | 3.7±0.2 |
| 13G-96 | JJ-192 | 418 | 4~11 | 0.55 | 2.31 | 2.43 | 12.14±0.33 | 2.96±0.12 | 3.8 | 4.1±0.2 |
| 13G-97 | JJ-214 | 504 | 4~11 | 0.70 | 2.75 | 2.43 | 14.01±0.47 | 3.04±0.12 | 5.1 | 4.6±0.2 |
| 13G-98 | JJ-233 | 542 | 4~11 | 0.66 | 2.58 | 2.43 | 17.03±0.33 | 3.01±0.12 | 4.8 | 5.7±0.3 |
| 13G-99 | JJ-250 | 576 | 4~11 | 0.67 | 2.27 | 2.30 | 18.72±0.25 | 2.85±0.11 | 7.2 | 6.6±0.3 |

**Table S2.** Radiocarbon ages of sediment samples from the JJ Profile, Mu Us Sandy Lands (*5*).

| Lab  no. | Sample  no. | Depth (cm) | Sediment  type | Dating material | PMC  (%) | ^14^Cage (Cal yr BP) | Cal yr BP (2σ) | Median age |
| --- | --- | --- | --- | --- | --- | --- | --- | --- |
| LUG12-173 | JJ296 | 698 | paleosol | Bulk organic | 45.75±0.63 | 6313±92 | 7005~7423 | 7214 |

**Table S3.** Microcharcoal concentrations data with different shapes and sizes of sediment samples from the JJ Profile, Mu Us Sandy Lands (unit: grains·g^-1^).

| Depth (cm) | Total PC | Total MC | MC_R_ | | | | MC_L_ | | | |
| --- | --- | --- | --- | --- | --- | --- | --- | --- | --- | --- |
|  |  |  | < 30 µm | 30-50 µm | 50-100 µm | > 100 µm | < 30 µm | 30-50 µm | 50-100 µm | > 100 µm |
| 0 | 3912 | 37051 | 11123 | 7287 | 2685 | 77 | 7671 | 4603 | 3298 | 307 |
| 8 | 1806 | 63067 | 19346 | 14187 | 1290 | 387 | 11736 | 9028 | 6449 | 645 |
| 16 | 141 | 3587 | 979 | 643 | 459 | 31 | 459 | 490 | 428 | 98 |
| 24 | 333 | 2755 | 865 | 546 | 260 | 0 | 406 | 446 | 213 | 20 |
| 32 | 459 | 1990 | 722 | 335 | 88 | 0 | 335 | 309 | 175 | 26 |
| 40 | 166 | 1525 | 477 | 256 | 105 | 0 | 281 | 251 | 140 | 15 |
| 48 | 68 | 1153 | 497 | 143 | 24 | 0 | 227 | 163 | 87 | 12 |
| 56 | 92 | 958 | 420 | 147 | 21 | 0 | 177 | 118 | 71 | 4 |
| 64 | 65 | 1401 | 524 | 284 | 61 | 0 | 227 | 196 | 100 | 9 |
| 72 | 93 | 1315 | 636 | 103 | 34 | 0 | 298 | 176 | 59 | 10 |
| 80 | 366 | 2121 | 913 | 235 | 55 | 0 | 399 | 306 | 208 | 5 |
| 88 | 197 | 1370 | 619 | 202 | 18 | 0 | 298 | 183 | 37 | 14 |
| 96 | 164 | 1268 | 504 | 180 | 55 | 0 | 256 | 231 | 38 | 4 |
| 104 | 109 | 1422 | 543 | 283 | 52 | 0 | 236 | 213 | 90 | 5 |
| 112 | 177 | 531 | 228 | 63 | 12 | 0 | 142 | 59 | 28 | 0 |
| 120 | 42 | 713 | 372 | 56 | 0 | 0 | 198 | 63 | 21 | 3 |
| 128 | 12 | 849 | 343 | 87 | 12 | 0 | 281 | 94 | 25 | 6 |
| 136 | 44 | 593 | 278 | 33 | 7 | 0 | 185 | 70 | 19 | 0 |
| 144 | 91 | 753 | 389 | 58 | 8 | 4 | 195 | 70 | 25 | 4 |
| 152 | 48 | 931 | 385 | 121 | 18 | 0 | 220 | 128 | 55 | 4 |
| 160 | 30 | 1156 | 581 | 81 | 30 | 0 | 288 | 141 | 30 | 5 |
| 168 | 72 | 1131 | 521 | 76 | 4 | 0 | 345 | 136 | 40 | 8 |
| 176 | 48 | 713 | 378 | 40 | 9 | 0 | 220 | 53 | 13 | 0 |
| 184 | 19 | 368 | 199 | 42 | 4 | 0 | 80 | 27 | 15 | 0 |
| 192 | 53 | 703 | 393 | 34 | 4 | 0 | 199 | 50 | 19 | 4 |
| 200 | 25 | 691 | 356 | 63 | 17 | 0 | 180 | 63 | 13 | 0 |
| 208 | 34 | 733 | 360 | 47 | 13 | 0 | 225 | 72 | 17 | 0 |
| 216 | 20 | 796 | 408 | 95 | 16 | 0 | 170 | 99 | 8 | 0 |
| 224 | 14 | 968 | 477 | 68 | 5 | 0 | 318 | 82 | 9 | 9 |
| 232 | 48 | 1378 | 510 | 171 | 64 | 0 | 315 | 211 | 104 | 4 |
| 240 | 55 | 1028 | 438 | 118 | 38 | 0 | 253 | 114 | 55 | 13 |
| 244 | 148 | 1316 | 421 | 278 | 39 | 0 | 261 | 234 | 83 | 0 |
| 248 | 139 | 1334 | 504 | 206 | 34 | 5 | 288 | 192 | 86 | 19 |
| 256 | 234 | 1316 | 570 | 144 | 37 | 5 | 336 | 181 | 32 | 11 |
| 260 | 208 | 1523 | 584 | 216 | 56 | 0 | 324 | 264 | 65 | 13 |
| 264 | 104 | 1059 | 456 | 161 | 33 | 0 | 247 | 109 | 47 | 5 |
| 272 | 63 | 1025 | 526 | 126 | 8 | 0 | 255 | 94 | 12 | 4 |
| 276 | 42 | 1660 | 764 | 170 | 55 | 0 | 391 | 221 | 55 | 4 |
| 280 | 1429 | 1627 | 798 | 175 | 23 | 0 | 395 | 175 | 53 | 8 |
| 284 | 704 | 2072 | 1016 | 235 | 31 | 0 | 508 | 203 | 70 | 8 |
| 288 | 520 | 2836 | 1320 | 332 | 91 | 0 | 671 | 317 | 83 | 23 |
| 292 | 177 | 2269 | 998 | 163 | 22 | 0 | 665 | 318 | 89 | 15 |
| 296 | 456 | 3122 | 1549 | 359 | 33 | 0 | 734 | 359 | 82 | 8 |
| 300 | 282 | 1518 | 690 | 207 | 13 | 0 | 376 | 138 | 82 | 13 |
| 304 | 119 | 993 | 477 | 103 | 8 | 0 | 199 | 167 | 40 | 0 |
| 308 | 127 | 2433 | 999 | 291 | 18 | 0 | 518 | 427 | 182 | 0 |
| 312 | 194 | 2537 | 927 | 270 | 118 | 0 | 548 | 472 | 194 | 8 |
| 316 | 59 | 2162 | 854 | 260 | 22 | 0 | 520 | 446 | 52 | 7 |
| 320 | 115 | 2335 | 841 | 280 | 41 | 0 | 619 | 437 | 107 | 8 |
| 328 | 120 | 964 | 466 | 66 | 4 | 0 | 272 | 136 | 16 | 4 |
| 332 | 25 | 926 | 399 | 102 | 18 | 0 | 254 | 109 | 40 | 4 |
| 336 | 66 | 1088 | 493 | 74 | 21 | 0 | 349 | 131 | 16 | 4 |
| 340 | 55 | 789 | 420 | 84 | 4 | 0 | 176 | 71 | 29 | 4 |
| 344 | 58 | 1202 | 584 | 136 | 5 | 0 | 302 | 122 | 49 | 5 |
| 348 | 74 | 1252 | 589 | 161 | 4 | 0 | 284 | 153 | 52 | 9 |
| 352 | 79 | 1214 | 523 | 126 | 10 | 0 | 340 | 168 | 42 | 5 |
| 356 | 26 | 1455 | 723 | 74 | 4 | 0 | 450 | 186 | 13 | 4 |
| 360 | 72 | 1403 | 725 | 76 | 10 | 0 | 406 | 134 | 48 | 5 |
| 364 | 17 | 901 | 491 | 51 | 9 | 0 | 278 | 60 | 9 | 4 |
| 368 | 23 | 1023 | 199 | 137 | 55 | 0 | 632 | 390 | 199 | 137 |
| 372 | 47 | 1023 | 484 | 109 | 39 | 0 | 199 | 137 | 55 | 0 |
| 380 | 10 | 814 | 373 | 51 | 10 | 0 | 221 | 126 | 34 | 0 |
| 388 | 18 | 1595 | 757 | 167 | 9 | 0 | 401 | 225 | 27 | 9 |
| 396 | 54 | 1744 | 822 | 163 | 8 | 0 | 488 | 202 | 54 | 8 |
| 400 | 42 | 1478 | 633 | 193 | 12 | 0 | 362 | 211 | 66 | 0 |
| 404 | 107 | 1449 | 692 | 207 | 21 | 0 | 264 | 228 | 36 | 0 |
| 408 | 89 | 1834 | 683 | 260 | 45 | 0 | 453 | 297 | 82 | 15 |
| 412 | 230 | 1336 | 729 | 77 | 31 | 0 | 261 | 161 | 69 | 8 |
| 420 | 38 | 800 | 431 | 67 | 17 | 0 | 197 | 63 | 21 | 4 |
| 424 | 33 | 1042 | 554 | 70 | 12 | 0 | 267 | 103 | 33 | 4 |
| 432 | 114 | 1521 | 725 | 156 | 21 | 0 | 412 | 171 | 28 | 7 |
| 440 | 26 | 411 | 233 | 22 | 4 | 0 | 115 | 30 | 4 | 4 |
| 448 | 7 | 406 | 200 | 47 | 14 | 0 | 91 | 41 | 10 | 3 |
| 456 | 51 | 604 | 314 | 61 | 10 | 0 | 140 | 48 | 31 | 0 |
| 464 | 19 | 794 | 457 | 45 | 15 | 0 | 197 | 60 | 15 | 4 |
| 472 | 22 | 606 | 311 | 35 | 3 | 3 | 163 | 63 | 25 | 3 |
| 480 | 21 | 627 | 355 | 34 | 14 | 0 | 186 | 31 | 7 | 0 |
| 488 | 24 | 627 | 326 | 48 | 4 | 0 | 183 | 64 | 0 | 4 |
| 496 | 10 | 352 | 192 | 32 | 6 | 0 | 90 | 29 | 0 | 3 |
| 500 | 35 | 724 | 364 | 105 | 12 | 0 | 194 | 46 | 0 | 4 |
| 504 | 34 | 955 | 492 | 86 | 4 | 0 | 283 | 75 | 11 | 4 |
| 508 | 16 | 1597 | 620 | 64 | 8 | 8 | 588 | 207 | 87 | 16 |
| 512 | 26 | 1555 | 453 | 98 | 7 | 0 | 512 | 276 | 197 | 13 |
| 516 | 9 | 1359 | 450 | 138 | 0 | 0 | 519 | 199 | 43 | 9 |
| 520 | 31 | 1884 | 628 | 212 | 8 | 0 | 589 | 369 | 78 | 0 |
| 524 | 8 | 1381 | 513 | 187 | 19 | 0 | 345 | 243 | 75 | 0 |
| 528 | 32 | 1897 | 577 | 115 | 19 | 6 | 609 | 436 | 122 | 13 |
| 532 | 26 | 1051 | 368 | 70 | 0 | 0 | 350 | 237 | 26 | 0 |
| 536 | 95 | 1389 | 429 | 80 | 15 | 0 | 378 | 371 | 109 | 7 |
| 540 | 67 | 2339 | 766 | 261 | 17 | 0 | 741 | 463 | 84 | 8 |
| 544 | 50 | 2156 | 743 | 123 | 22 | 0 | 649 | 461 | 144 | 14 |
| 548 | 108 | 1938 | 768 | 172 | 0 | 7 | 675 | 215 | 86 | 14 |
| 552 | 130 | 1530 | 545 | 112 | 17 | 0 | 432 | 346 | 78 | 0 |
| 556 | 30 | 646 | 230 | 22 | 7 | 0 | 267 | 104 | 15 | 0 |
| 560 | 32 | 1058 | 379 | 39 | 0 | 0 | 426 | 158 | 47 | 8 |
| 564 | 27 | 4046 | 1708 | 378 | 36 | 0 | 1259 | 512 | 135 | 18 |
| 568 | 24 | 1031 | 331 | 47 | 0 | 0 | 417 | 197 | 31 | 8 |
| 572 | 785 | 1124 | 509 | 134 | 28 | 0 | 247 | 170 | 35 | 0 |
| 576 | 500 | 1471 | 709 | 157 | 15 | 0 | 284 | 217 | 82 | 7 |
| 580 | 84 | 1977 | 673 | 76 | 0 | 0 | 841 | 278 | 101 | 8 |
| 584 | 79 | 1966 | 695 | 79 | 16 | 0 | 711 | 395 | 71 | 0 |
| 588 | 8 | 3810 | 1563 | 118 | 8 | 0 | 1267 | 760 | 84 | 8 |
| 592 | 77 | 4852 | 1617 | 187 | 68 | 0 | 1660 | 936 | 358 | 26 |
| 596 | 63 | 2776 | 706 | 141 | 0 | 0 | 1255 | 588 | 86 | 0 |
| 600 | 134 | 3558 | 1125 | 246 | 35 | 0 | 1125 | 844 | 176 | 7 |
| 604 | 122 | 3599 | 1002 | 105 | 35 | 0 | 1481 | 871 | 105 | 0 |
| 608 | 103 | 4529 | 1383 | 198 | 24 | 0 | 1660 | 1028 | 221 | 16 |
| 612 | 129 | 10447 | 5895 | 692 | 132 | 9 | 1848 | 1154 | 630 | 87 |
| 616 | 320 | 14370 | 8235 | 584 | 28 | 0 | 3102 | 2087 | 181 | 153 |
| 620 | 62 | 2813 | 1084 | 223 | 25 | 0 | 836 | 527 | 118 | 0 |
| 624 | 170 | 3710 | 1822 | 259 | 16 | 0 | 972 | 445 | 186 | 8 |
| 628 | 133 | 5135 | 2611 | 366 | 60 | 7 | 1399 | 386 | 253 | 53 |
| 632 | 177 | 3774 | 1853 | 234 | 114 | 0 | 969 | 342 | 228 | 34 |
| 636 | 106 | 4146 | 1694 | 229 | 9 | 0 | 1632 | 450 | 132 | 0 |
| 640 | 77 | 3018 | 1152 | 177 | 61 | 0 | 845 | 576 | 192 | 15 |
| 640 | 89 | 2442 | 933 | 154 | 32 | 0 | 730 | 406 | 162 | 24 |
| 646 | 154 | 7895 | 3299 | 814 | 132 | 0 | 2089 | 1232 | 286 | 44 |
| 648 | 82 | 1764 | 783 | 16 | 0 | 0 | 577 | 346 | 41 | 0 |
| 650 | 126 | 5758 | 2674 | 551 | 110 | 0 | 1447 | 724 | 236 | 16 |
| 652 | 94 | 4231 | 1966 | 31 | 8 | 0 | 1927 | 204 | 94 | 0 |
| 656 | 153 | 5995 | 2390 | 451 | 8 | 0 | 2132 | 805 | 209 | 0 |
| 660 | 141 | 4603 | 2036 | 249 | 0 | 0 | 1662 | 540 | 116 | 0 |
| 664 | 136 | 4839 | 1816 | 118 | 64 | 0 | 1589 | 953 | 218 | 82 |
| 668 | 230 | 7968 | 3714 | 646 | 71 | 0 | 2122 | 929 | 371 | 115 |
| 672 | 74 | 2086 | 814 | 160 | 44 | 0 | 587 | 333 | 136 | 12 |
| 676 | 88 | 2057 | 703 | 44 | 0 | 0 | 747 | 404 | 158 | 0 |
| 680 | 160 | 3020 | 1262 | 185 | 34 | 0 | 757 | 505 | 252 | 25 |
| 684 | 96 | 3299 | 1204 | 153 | 64 | 8 | 963 | 642 | 265 | 0 |
| 688 | 85 | 1974 | 602 | 163 | 35 | 0 | 602 | 354 | 191 | 28 |
| 692 | 90 | 3154 | 1030 | 188 | 18 | 0 | 1254 | 448 | 206 | 9 |
| 696 | 56 | 2218 | 702 | 84 | 9 | 0 | 795 | 449 | 140 | 37 |
| 700 | 108 | 2232 | 693 | 85 | 8 | 0 | 770 | 462 | 192 | 23 |
| 704 | 50 | 1047 | 332 | 91 | 0 | 0 | 415 | 174 | 33 | 0 |
| 708 | 27 | 2007 | 622 | 53 | 27 | 0 | 710 | 444 | 124 | 27 |
| 712 | 72 | 1357 | 602 | 27 | 0 | 0 | 432 | 270 | 27 | 0 |
| 716 | 23 | 319 | 106 | 14 | 5 | 0 | 111 | 74 | 9 | 0 |
| 720 | 16 | 283 | 77 | 26 | 19 | 0 | 97 | 58 | 6 | 0 |
| 724 | 26 | 970 | 372 | 52 | 5 | 0 | 367 | 147 | 26 | 0 |
| 728 | 7 | 506 | 221 | 14 | 7 | 0 | 164 | 85 | 14 | 0 |
| 732 | 6 | 798 | 301 | 38 | 0 | 0 | 345 | 82 | 25 | 6 |
| 740 | 86 | 580 | 316 | 27 | 10 | 0 | 151 | 55 | 17 | 3 |

**Supplementary Reference**

S1. Maher, L. J. Statistics for microfossil concentration measurements employing samples spiked with marker grains. *Rev. Palaeobot. Palynol.* 32, 153-191 (1981).
